# Evaluation of the efficacy of plan adaptation in stereotactic body proton therapy for pancreatic cancer

**DOI:** 10.1002/acm2.70644

**Published:** 2026-05-28

**Authors:** Yuto Matsuo, Yusuke Uchinami, Norio Katoh, Takayuki Hashimoto, Hidefumi Aoyama, Keiji Nakazato, Taeko Matsuura, Naoki Miyamoto, Seishin Takao

**Affiliations:** ^1^ Graduate School of Biomedical Science and Engineering Hokkaido University Sapporo Japan; ^2^ Department of Medical Physics Hokkaido University Hospital Sapporo Japan; ^3^ Department of Radiation Oncology Hokkaido University Hospital Sapporo Japan; ^4^ Global Center for Biomedical Science and Engineering Faculty for Medicine Hokkaido University Sapporo Japan; ^5^ Department of Radiation Oncology Faculty of Medicine Hokkaido University Sapporo Japan; ^6^ Faculty of Engineering Hokkaido University Sapporo Japan

**Keywords:** daily dose evaluation, pancreatic cancer, plan adaptation, stereotactic body proton therapy

## Abstract

**Purpose:**

This study aimed to quantitatively evaluate the efficacy of plan adaptation in stereotactic body proton therapy (SBPT) for pancreatic cancer using daily CT‐based dose evaluation, investigate the appropriate adaptation frequency.

**Methods:**

This retrospective planning study included 10 patients previously treated with X‐ray stereotactic body radiation therapy for pancreatic cancer. An initial SBPT plan was created for each patient, using robust optimization with two posterior oblique beams. The prescribed dose was D90 ≥ 40 Gy(RBE) to the CTV_EVAL (the CTV excluding the gastrointestinal [GI] tract plus a 5‐mm margin), delivered in five fractions. Daily dose evaluation (DDE) was performed using five daily CT (dCT) images per patient. An adaptive plan (AP) was generated and applied to subsequent fractions if target coverage or organs at‐risk (OARs) constraints were not met; otherwise, the initial plan (IP) was employed. Non‐adaptation (NA) and daily adaptation (DA) strategies were compared in terms of dose‐volume metrics, accumulated doses, tumor control probability (TCP), and normal tissue complication probability (NTCP).

**Results:**

Across 50 fractions, plan adaptation was necessary in 21 fractions (42%), with a mean of 2.1 adaptations per patient. DA significantly improved target coverage on DDE compared with NA (*p* < 0.05). Doses to the OARs, particularly the GI tract, were significantly reduced with DA. In the accumulated dose, DA led to a significantly higher D90 of CTV_EVAL (*p* < 0.05), whereas doses to the OARs demonstrated no significant differences between strategies. TCP was significantly improved with DA, whereas NTCP demonstrated no significant differences for any OARs.

**Conclusions:**

The DA strategy, according to DDE, improved target dose coverage while maintaining or reducing doses to the OARs. These findings indicate that appropriately frequent plan adaptation—an average of 2.1 adaptations per patient—may improve SBPT safety and efficacy for pancreatic cancer.

## 1 INTRODUCTION

Pancreatic cancer is associated with a low 5‐year survival rate of less than 10% and ranks as the seventh leading cause of cancer‐related deaths globally.^1^, ^2^ Multidisciplinary treatment that combines surgery, chemotherapy, and radiotherapy has been adopted to improve treatment outcomes; however, the recurrence rate remains high, indicating a very poor prognosis.^3^, ^4^

Radiosensitive organs, including the stomach, duodenum, and bowel, surround the pancreas, which limits the ability to deliver a sufficiently high dose to the tumor. Consequently, conventional radiotherapy has been greatly ineffective. Intensity‐modulated radiation therapy has enabled both dose reduction to the organs at risk (OARs) and a high‐dose delivery to the tumor.^5^ Furthermore, stereotactic body radiation therapy (SBRT), which mitigates tumor respiratory motion through high‐precision patient positioning and treatment beam control, incorporating image‐guided radiation therapy and various motion management techniques, including respiratory gating and tumor tracking, has enabled smaller margins around the target than previously achieved.^6^, ^7^ Further, SBRT facilitates hypo‐fractionated irradiation, characterized by fewer fractions and higher doses per fraction, thereby potentially providing a higher biologically effective dose to the tumor and improving local control rates compared with conventional methods.

However, the position and shape of the gastrointestinal (GI) tract are subject to daily variations due to gas and other factors, requiring careful thought regarding potential unexpected high doses to the GI tract.^8^, ^9^ Daily adaptive planning, accounting for these daily changes in body shape and anatomical organ positions, provides a solution.^10^, ^11^, ^12^, ^13^

Recent advancements include stereotactic body proton therapy (SBPT), which aims to reduce doses to the OARs more effectively than SBRT. Due to their unique depth–dose characteristic known as the Bragg Peak, proton beams enable a more concentrated dose distribution to the tumor compared with X‐ray beams. Especially in pancreatic cancer, previous studies have revealed serious GI toxicities, including intestinal bleeding, after SBPT without daily adaptation (DA).^1^ Plan adaptation has been well established as essential in SBRT;^14^ however, its role in SBPT remains unclear. Furthermore, a recent study on plan adaptation has demonstrated that the implementation of adaptive radiotherapy markedly elevated the overall workload compared with conventional radiotherapy, leading to a substantial increase in personnel demands and the burden on clinical workflow across multiple professional disciplines.^15^ Hence, this study aimed to quantitatively evaluate the efficacy of plan adaptation in SBPT for pancreatic cancer and to investigate the appropriate frequency of plan adaptation.

## 2 METHODS

### 2.1 Patient characteristics

This retrospective planning study included 10 patients who underwent X‐ray SBRT for pancreatic cancer at Hokkaido University Hospital from September 2020 to June 2022. Our institutional review board (IRB number: 019–0397) approved the study. Eligible patients aged 51–90 years (median, 78.5 years), comprising 8 males and 2 females. Gating irradiation with real time tumor tracking is employed at our hospital to mitigate the reduction of dose uniformity due to respiratory motion and prevent unnecessary excess dose to surrounding normal tissues. Patients underwent fiducial marker implantation near the tumor before treatment planning computed tomography (CT) imaging.^7^, ^16^ Table 1 shows patient details.

**TABLE 1:** Patients’ characteristics.

Patient	Age	Sex	Tumor location	GTV size [cc]	CTV size [cc]
1	90	F	Body	5.0	7.0
2	88	M	Body	6.8	10.1
3	73	M	Body	2.0	2.8
4	78	M	Body/Tail	13.3	15.2
5	71	M	Head	2.1	3.7
6	79	M	Head	1.0	1.8
7	81	M	Head	7.9	11.1
8	51	M	Body/Tail	3.5	4.9
9	85	F	Head	3.8	5.2
10	76	M	Head	15.1	18.7

Abbreviations: M, male; F, female; Body, pancreatic body; Tail, pancreatic tail; Head, pancreatic head; GTV, gross tumor volume; CTV, clinical target volume.

### 2.2 CT data acquisition

Planning CT (pCT) images were acquired based on our clinical protocol using a SOMATOM Confidence RT Pro (Siemens Healthineers, Erlangen, Germany) scanner.^7^ After a minimum 6‐h fast, patients were immobilized with a vacuum cushion, and both plain and contrast‐enhanced CT scans with a 2‐mm slice thickness were obtained during breath‐hold at end‐exhalation and utilized as pCT images. However, treatment beams were delivered with respiratory gating at the end‐exhalation phase under free‐breathing conditions; thus, confirming that the marker position at end‐exhalation did not significantly deviate between breath‐hold and natural exhalation was essential. Therefore, four‐dimensional CT (4DCT) employing a real time position management system (Varian Medical Systems, Palo Alto, CA) was concurrently performed. Experienced radiation oncologists reviewed the pCT images to verify that the marker position relative to the bone structure in the cranio‐caudal direction was within two slices (approximately 4 mm) compared with that in the exhalation phase of the 4DCT.

### 2.3 Volume delineation of target and OARs

Experienced radiation oncologists delineated volumes of target and OARs on the pCT images. The gross tumor volume (GTV) was contoured on plane CT images with reference to contrast‐enhanced CT and positron emission tomography images. Major vessels within 5 mm of the GTV refer to the tumor‐vessel interface (TVI), and the clinical target volume (CTV) was defined as the combination of the GTV and TVI.^17^ CTV_EVAL refers to the CTV excluding the GI tract plus a 5‐mm margin, to address the potential conflict between CTV coverage and OAR dose constraints when the CTV and GI tract are in proximity. GI tracts (stomach, duodenum, small bowel, and large bowel) were defined as OARs, and a 5‐mm planning at‐risk volume (PRV) margin was added to each OAR (stomach_PRV, duodenum_PRV, small bowel_PRV, and large bowel_PRV).

### 2.4 SBPT planning simulation

In this study, all simulation plans were generated assuming SBPT employing a commercial treatment planning system, VQA (Hitachi, Ltd., Japan), commissioned for the PROBEAT‐RT system (Hitachi, Ltd., Japan). Two oblique beam angles—150° and 210°—were used^18^, ^19^ and optimized with multi‐field optimization for CTV_EVAL. In X‐ray therapy, optimization is performed for the planning target volume, which is created by expanding the CTV using a geometric margin to compensate for internal motion and setup error. However, this approach is insufficient for proton therapy planning.^20^, ^21^ Consistent with our clinical protocol, we performed robust optimization for the CTV_EVAL utilizing a worst‐case scenario that considered a 5‐mm setup error and a ± 3.5% range uncertainty associated with patient stopping power ratio estimation.^22^ The dose prescription was D90 (minimum dose covering 90% of the volume) of at least 40 Gy(relative biological effectiveness [RBE]) for the CTV_EVAL in five fractions, assuming an RBE of 1.1. Dose constraints for OARs were based on the report by Oars et al.,^17^ specifying that the volume receiving more than 33 Gy(RBE) (V33) for the stomach, duodenum, small bowel, and large bowel should be less than 0.5 cc. The volume receiving more than 38 Gy(RBE) (V38) should be less than 0.5 cc for each PRV (stomach_PRV, duodenum_PRV, small bowel_PRV, and large bowel_PRV). These constraints were defined with reference to photon SBRT dose constraints for pancreatic cancer. Table 2 shows details. The dose constraints for OARs were given the highest priority during plan optimization. Predefined acceptable criteria were applied when the prescribed planning objectives could not be simultaneously satisfied due to anatomical proximity between the target and adjacent GI OARs. In such cases, an acceptable CTV coverage range of 30 Gy(RBE) ≤ D99 < 33 Gy(RBE) was defined, and optimization was performed to achieve the highest possible target coverage within this range while maintaining the OAR dose constraints. Experienced radiation oncologists approved the generated plan, defined as the initial plan (IP).

**TABLE 2:** Dose constraints for SBPT simulation.

ROI	Criteria	Acceptable criteria
CTV_EVAL	D_90_ ≥ 40 Gy(RBE)	36 Gy(RBE)≤ D_90_ < 40 Gy(RBE)
	48 Gy(RBE)≤ D_2_ ≤ 50 Gy(RBE)	N/A
CTV	D_99_ > 33 Gy(RBE)	30 Gy(RBE)≤ D_99_ < 33 Gy(RBE)
GTV	D_50_ ≥ 40 Gy(RBE)	D_2_ ≥ 40 Gy(RBE)
Stomach	V_33_ <0.5 cc	N/A
Duodenum		
Small bowel		
Large bowel		
each OAR_PRV	V_38_ < 0.5 cc	

Abbreviations: SBPT, stereotactic body proton therapy; ROI, region of interest; CTV, clinical target volume; GTV, gross tumor volume; OARs, organ at risk; PRV, planning at‐risk volume; RBE, relative biological effectiveness. Dx refers to the dose received by X percent of the target volume at least; Vx refers to volumes irradiated X Gy(RBE) at least.

### 2.5 Daily dose evaluation (DDE) and plan adaptation strategy

**FIGURE 1:**
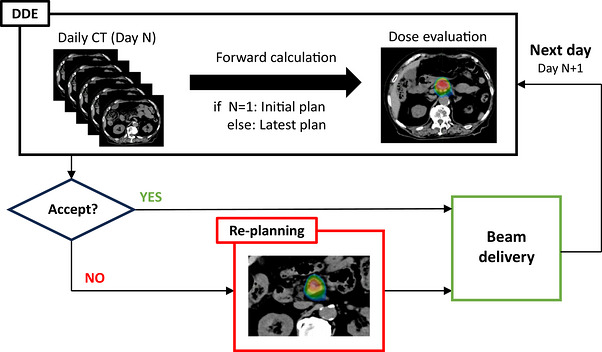
Overview of daily dose evaluation and plan adaptation.

DDE was performed on daily CT (dCT) images acquired in a dedicated CT room separate from the proton treatment room. The dCT images were obtained on each treatment day utilizing the following procedure. Rigid image registration (RIR) with six degrees of freedom was performed between the pCT and dCT images employing MIM Maestro software (MIM Software Inc., Cleveland, OH, USA) with bony references. The bone‐matched dCT images were translationally registered to align the marker positions with those on pCT images. At our institution, this registration procedure replicates the patient setup for real time image‐guided proton therapy (RGPT) for moving tumors.^23^ The regions of interest (ROIs) for assessment were transferred to the dCT images by deforming the ROIs on the pCT images utilizing deformable image registration (DIR). Experienced radiation oncologists reviewed and modified the contours of the target and OARs, as necessary. After virtually reproducing the daily patient setup on dCT images following the procedure described above, forward dose calculations were performed with an in‐house tool to evaluate the need for plan adaptation. Registration between the pCT and dCT was required to perform DDE. This functionality was not available in the TPS used in our facility; thus, in‐house software was used instead. This tool employs the same calculation algorithm as the VQA used for treatment planning. The 3D gamma analysis revealed that dose distributions calculated with the in‐house tool and VQA demonstrated over 99% agreement within a 1 mm/1% tolerance. The criteria for identifying plan adaptation were defined separately for OARs and the target. The criterion for OARs was that the V38 (the volume receiving more than 38 Gy[RBE]) of each OAR_PRV should be less than 0.5 cc. The criterion for the target was that the minimum dose covering 50% of the volume (D50) of the GTV should be 38 Gy(RBE) or higher. The geometry of CTV_EVAL may change daily based on the positional relationship between the tumor and adjacent GI organs; thus, the use of D90 of CTV_EVAL as an adaptation trigger overall could lead to fluctuations that do not necessarily reflect true target underdosage. Therefore, D50 of GTV was selected as the decision metric for DA because it's less sensitive to inter‐fractional GI organ displacement and was included in the original planning objectives. In this study, 38 Gy(RBE) (95% of the prescribed dose of 40 Gy[RBE]) was utilized as the threshold for triggering adaptive replanning. Further, the maximum dose delivered to a 0.5 cc volume within the organ of interest (D0.5) was calculated to evaluate doses to the OARs in more detail. The DA strategy simulated in this study is summarized as follows. On the first treatment day, DDE is performed using the IP to assess whether it meets the adaptation criteria described above. The IP is delivered if all criteria are satisfied; otherwise, an adaptive plan (AP) is generated and delivered. The latest delivered plan is then carried forward as the candidate plan for the next treatment day. DDE is performed using the candidate plan from the second fraction onward, and the need for adaptation is evaluated similarly. This process is repeated until the fifth fraction. Figure 1 illustrates the workflow of this DA approach. Conversely, the strategy of delivering the IP on all treatment days without DDE was defined as a non‐adaptation (NA) method.

### 2.6 Evaluation of the efficacy of the adaptive strategy

This study investigated the number of treatment fractions, in which AP was applied among the five fractions for each of the 10 patients, to evaluate the efficacy of the proposed adaptive strategy. Further, accumulation based on DIR was performed for all five fractions with reference to pCT images, and doses to the target and OARs were compared between the NA and DA strategies.

### 2.7 TCP/NTCP models

Tumor control probability (TCP) and normal tissue complication probability (NTCP) generated by the NA and DA strategy were calculated. To calculate TCP, we referred to the paper by Mahadevan et al.,^24^ converting doses to a three‐fraction equivalent using the linear‐quadratic (LQ) model with α/β of 10 Gy. Logistic modeling via maximum likelihood parameter fitting of the resulting three‐fraction equivalent doses was then performed employing the following TCP formula:

(1) $$TCP=\frac{1}{\left\{1+{\left(TCD_{50}/D\right)}^{4g_{50}}\right\}}$$

where $TCD_{50}$ is the dose required when the TCP is 50%, and *D* is the prescription dose, and $g_{50}$ is the slope parameter. The D90 of CTV_EVAL was utilized to calculate the TCP.

Before the NTCP calculation, the LQ model with α/β of 4 (for the stomach, duodenum, small bowel, and stomach–duodenum [stoduo]) was used to convert the cumulative physical dose into an equivalent dose in 2 Gy(RBE) per fraction (EQD2).^25^ “Stoduo” represented the combined stomach and duodenum. For NTCP calculation, we referred to the papers by Pan et al, Burman et al, and Holyoake et al, as Table 3 lists.^26^, ^27^, ^28^ We employed the Lyman–Kutcher–Burman (LKB) model for each GI tract endpoint, as follows:^29^, ^30^

(2) $$NTCP=\frac{1}{\sqrt{2\pi }}\;\int_{-\infty }^{t}e-\frac{x^{2}}{2}dx$$

where t is defined as

(3) $$t=\frac{gEUD-TD_{50}}{mTD_{50}}\;$$

and gEUD is

(4) $$gEUD={\left(\sum_{i}v_{i}D_{i}^{1/n}\right)}^{n}\;$$

and denotes the dose assuming uniform distribution across the volume, produces the same complication probability as the actual dose distribution represented by the summation.

**TABLE 3:** LKB model parameters used in the biological evaluation of the NA and DA strategies.

Gastrointestinal OAR (Reference)	TCD_50_ (Gy[RBE])	*m*	*n*	Endpoint
Stomach wall (Pan et al.)^26^	62	0.30	0.07	Gastric bleed
Stomach wall (Burman et al.)^25^	65	0.14	0.15	Ulceration/Perforation
Duodenum (Pan et al.)^26^	180	0.49	0.12	Gastric bleed
Duodenum (Holyoake et al.)^27^	299.1	0.51	0.193	Grade ≥3 GI toxicity
Small bowel loops (Burman et al.)^25^	55	0.16	0.15	Obstruction/Perforation
StoDuo (Pan et al.)^26^	52.5	0.35	0.21	Gastric bleed

Abbreviations: OAR, organ at risk; TCD_50_(Gy[RBE]), dose at which there is 50% chance of complication; m, slope of dose‐response curve; n, dose‐volume relationship

Variables $v_{i}$ and $D_{i}$ are the volume and dose of the *i*‐th bin of the dose‐volume histogram (DVH). $TCD_{50}$ is the dose that produces a 50% complication probability assuming uniform distribution across the organ. The variable *m* refers to the slope of the integral of the normal distribution, and *n* denotes whether the tissue is parallel or serial.

### 2.8 Statistical analysis

Wilcoxon signed‐rank tests were conducted to analyze significant differences in the DVH metrics of the target and OARs, and between the DA and NA groups in TCP/NTCP values, with a significance *p*‐value of 0.05. JMP PRO version 17.2 (SAS Institute, Cary, NC, USA) was used for statistical analysis.

## 3 RESULTS

### 3.1 Results of DDE and plan adaptation

**FIGURE 2:**
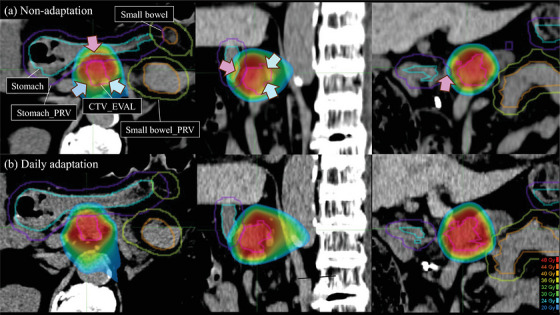
Representative dose distributions for (a) non‐adaptation and (b) daily adaptation on the same day (axial on the left, sagittal in the middle, and coronal on the right). The blue arrows represent cold spots within the target; the pink arrows denote hot spots within the OAR in the case of non‐adaptation.

Figure 2 illustrates representative dose distributions for NA and DA on the same day. An increase in the high‐dose region in the stomach and a reduction in the posterior part of the target were observed in the NA case, whereas DA improved the dose distribution.

**FIGURE 3:**
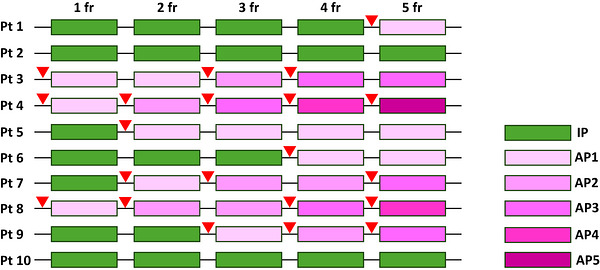
Delivered plan for each fraction in the simulation of daily adaptation. The legend is shown on the right. The red markers indicate the timing of plan adaptation. IP, initial plan; APn, *n*‐*th* adaptive plan.

Figure 3 illustrates the results of all 50 simulations of DA. The cell color represents the delivered plans according to the proposed daily adaptive strategy.

Based on DDE, plan adaptation was required in 21 out of 50 fractions. Only two patients completed the entire treatment utilizing the IP alone. The average number of plan adaptations was 2.1; 0 in 2 cases, 1 in 3 cases, 3 in 3 cases, 4 in 1 case, and 5 in 1 case. The number of fractions that deviated from the dose constraints was 3 in the stomach, 13 in stomach_PRV, 3 in duodenum_PRV, 2 in small bowel_PRV, 1 in large bowel, 3 in large bowel_PRV, and 3 in D50 for GTV. Overall, among the 21 adapted fractions, 4.8% (1 fraction) were triggered by D50 underdosage for GTV, 85.7% (18 fractions) by OAR constraint violations, and 9.5% (2 fractions) by both criteria. Plan adaptation was not required in 29 fractions.

**FIGURE 4:**
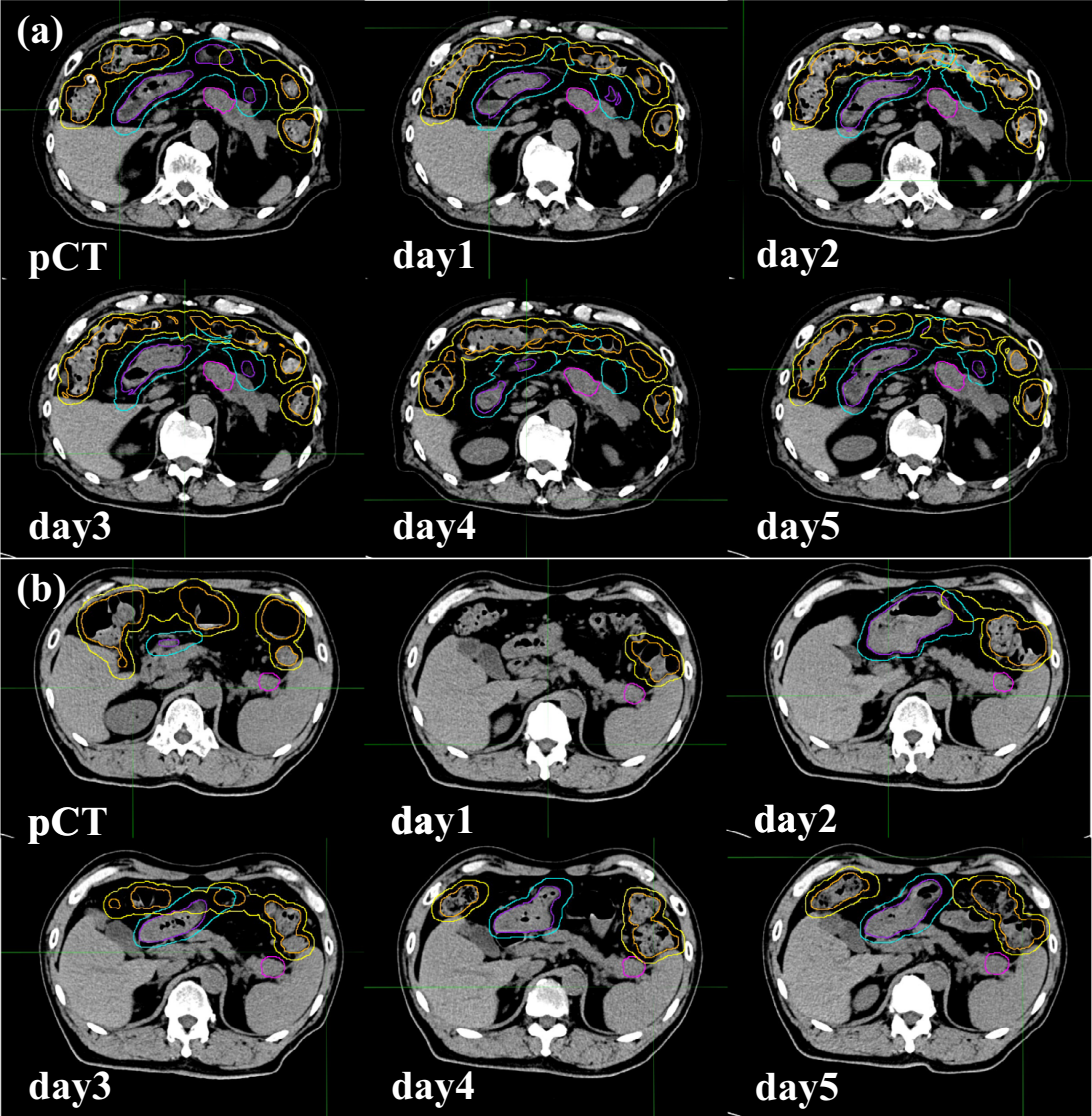
Daily positional changes of the GI tract and GTV for (a) patient 4 and (b) patient 8. For each patient, axial images near the tumor center on the pCT and on five treatment days are shown. The regions of interest (ROI) are displayed in the GTV (magenta), stomach (purple), stomach_PRV (cyan), large bowel (orange) and large bowel_PRV (yellow).

Figure 4 illustrates the daily positional changes of the GI tract and GTV for (a) patient 4 and (b) patient 8. For each patient, axial images near the tumor center on the pCT and on five treatment days are shown. Based on DDE, these daily anatomical variations required plan adaptation in five fractions for patient 4 and four fractions for patient 8.

### 3.2 Comparison of the DVH metrics and TCP/NTCP values

**FIGURE 5:**
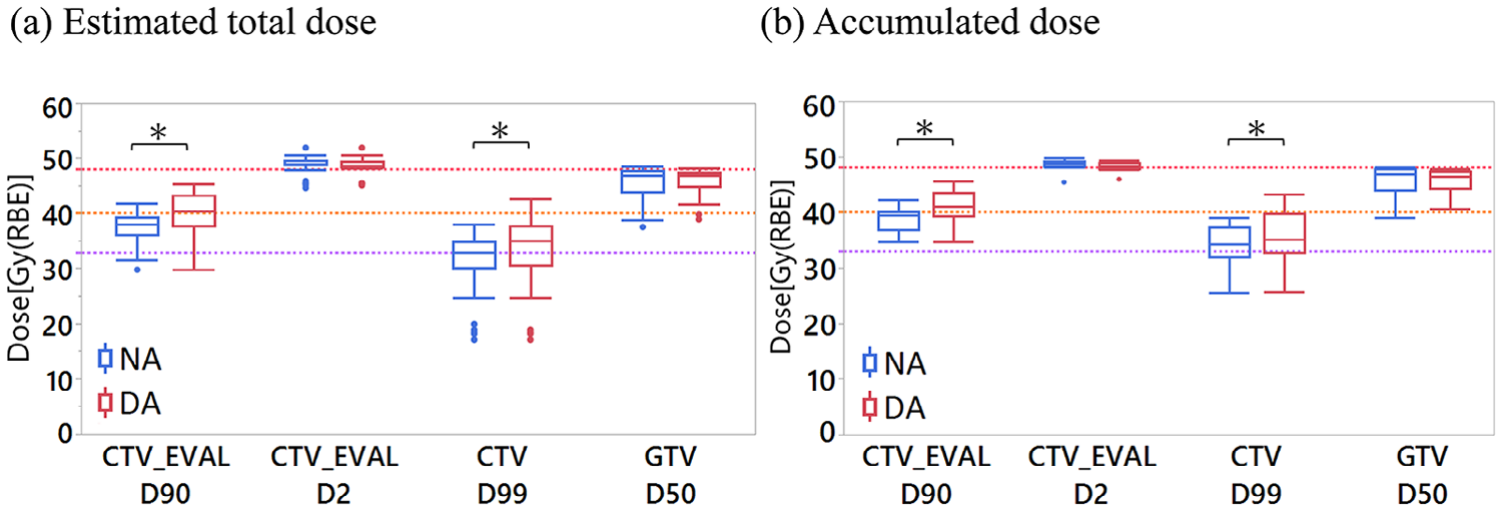
Comparison of DVH metrics in target between NA (blue) and DA (red) for (a) estimated total dose (*N* = 50) and (b) accumulated dose (*N* = 10). The red dashed line represents the criteria for D2 of CTV_EVAL, the orange dashed line for D90 of CTV_EVAL and D50 of GTV, and the purple dashed line for D99 of CTV for the target. The black dashed line represents the criteria for OARs and each OAR_PRV. The asterisks (^∗^) indicate statistically significant differences (*p* < 0.05) using the Wilcoxon signed‐rank test. Abbreviations: DX refers to the dose received by *X* percent of the target volume at least

**FIGURE 6:**
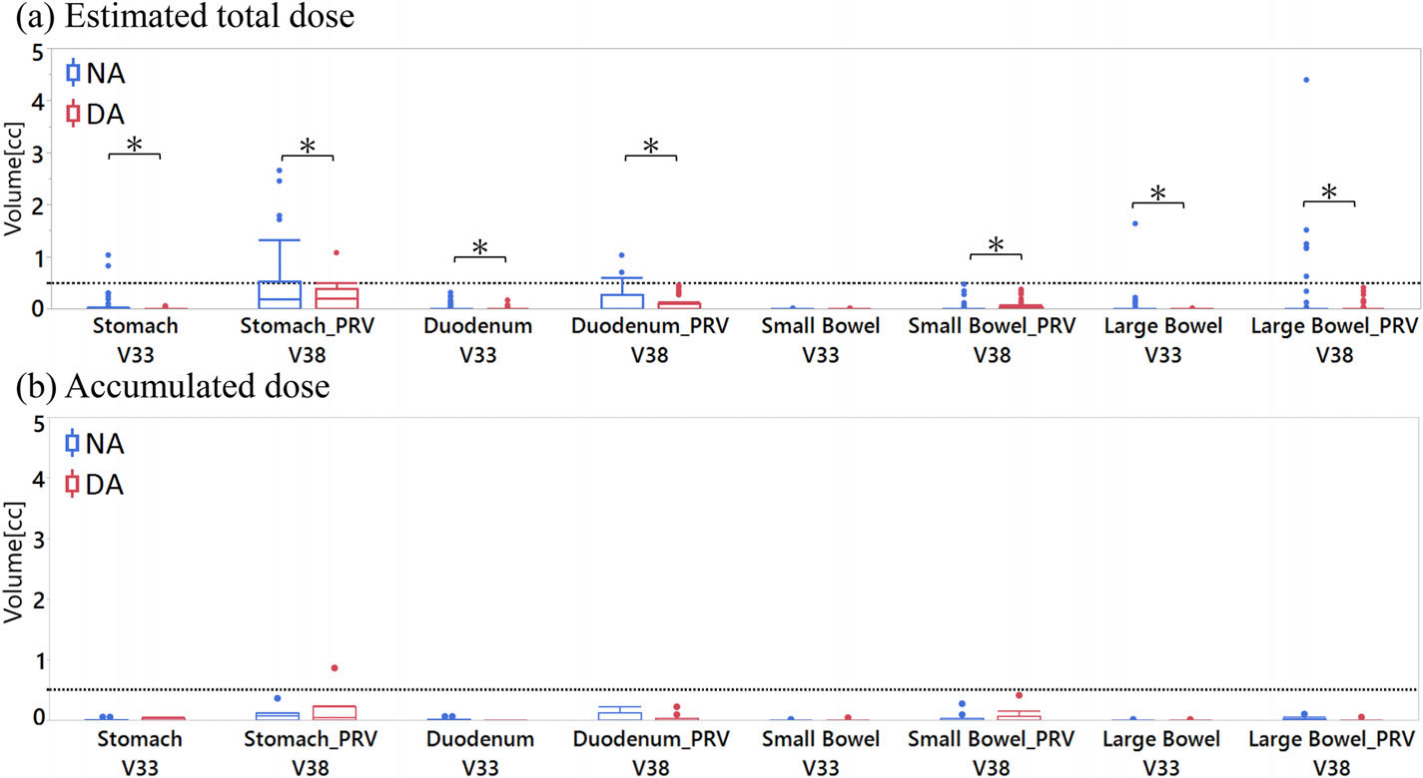
Comparison of DVH metrics in OARs between NA (blue) and DA (red) for (a) estimated total dose (*N* = 50) and (b) accumulated dose (*N* = 10). The black dashed line represents the criteria for OARs and each OAR_PRV. The asterisks (^∗^) indicate statistically significant differences (*p* < 0.05) using the Wilcoxon signed‐rank test. Abbreviations: VX refers to volumes irradiated X Gy(RBE) at least

Figures 5 and 6 illustrate the comparison of the DVH metrics in target and OARs between NA and DA. Figures 5a and 6a illustrate the results of the evaluation based on the estimated total dose calculated for each fraction (daily dose × 5 fractions, *N* = 50). The median (minimum–maximum range) of D90 of CTV_EVAL for NA and DA were 38.0 (29.7–41.8) Gy(RBE) and 40.4 (29.7–45.3) Gy(RBE), 32.9 (17.1–38.1) Gy(RBE), and 35.0 (17.1–42.6) Gy(RBE) for D99 of CTV, respectively. The dose coverage of the target was significantly improved with DA (*p *< 0.05). All DVH metrics for OARs were significantly lower except for V33 of small bowel and D0.5 for small bowel_PRV (*p *< 0.05). Table S1 summarizes other detailed results.

Figures 5b and 6b illustrate the results of the evaluation based on the accumulated doses (*N* = 10). The median (minimum–maximum range) of D90 of CTV_EVAL for NA and DA were 39.5 (34.7–42.2) Gy(RBE) and 41.1 (34.7–45.6) Gy(RBE), respectively. Further, the target dose was significantly improved with DA for the accumulated dose (*p *< 0.05). The doses to the OARs demonstrated no significant differences in any DVH metrics between NA and DA.

**FIGURE 7:**
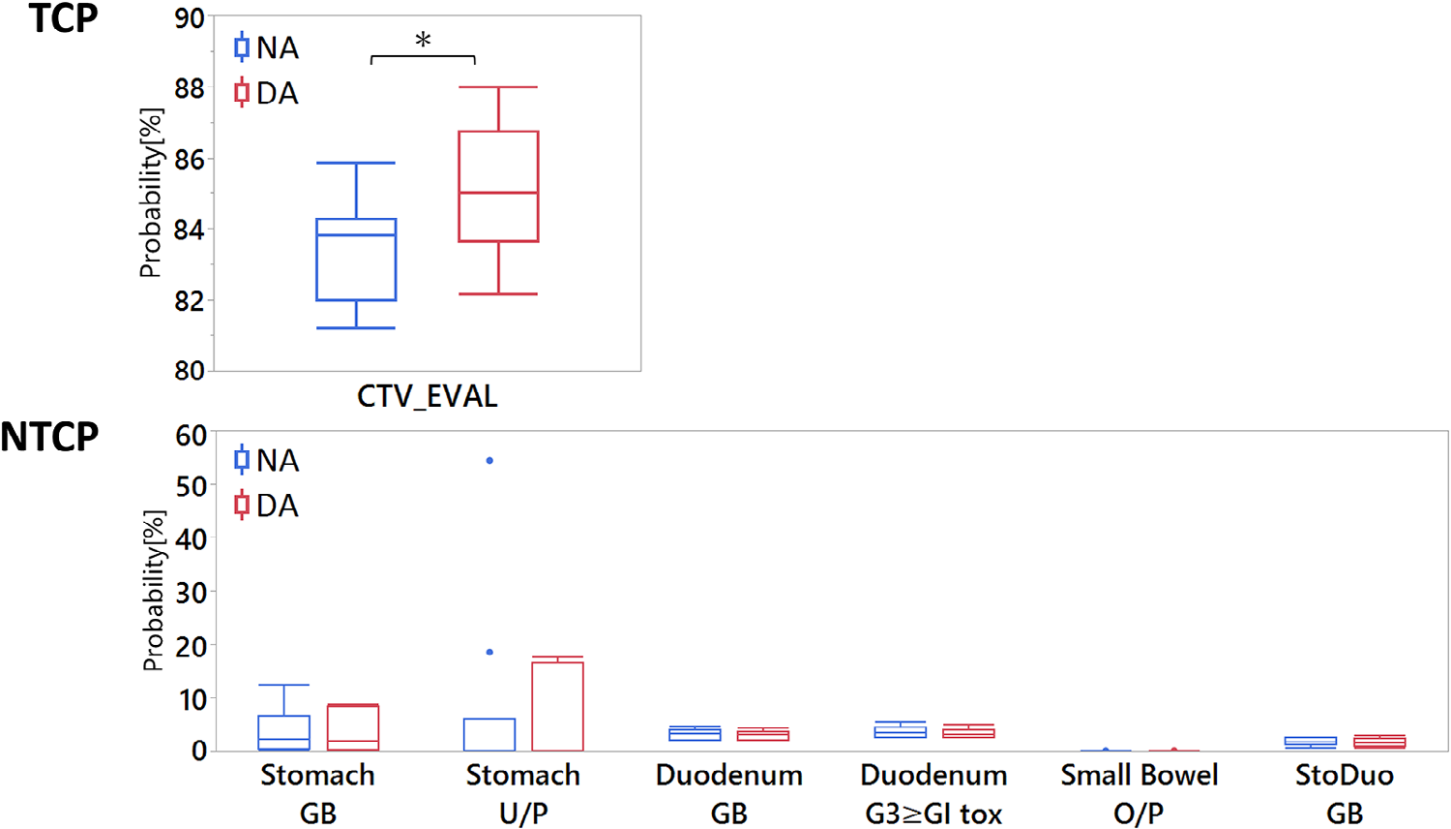
Comparison of tumor control probability (TCP) and normal tissue complication probability (NTCP) values between NA (blue) and DA (red) for accumulated dose (*N* = 10). The asterisk (^∗^) indicates statistically significant differences (*p* < 0.05) using the Wilcoxon signed‐rank test. StoDuo, combining of stomach and duodenum; U/P, ulceration/perforation; G3 ≥ GI tox, grade ≥ 3 GI toxicity; O/P, obstruction/perforation.

The TCP/NTCP values were calculated from the accumulated doses, and Figure 7 illustrates their comparison between NA and DA. The median (minimum–maximum range) of TCP for CTV_EVAL was 83.8% (81.2%–85.9%) for NA and 85.0% (82.2%–88.0%) for DA. TCP was significantly increased in the DA (*p *< 0.05). No significant differences in NTCP were observed between the NA and DA for all OARs.

## 4 DISCUSSION

This study quantitatively evaluated the efficacy of plan adaptation in SBPT for pancreatic cancer, utilizing DVH metrics and TCP/NTCP values. The results indicate that DDE using dCT images and replanning as necessary reduces the dose to surrounding OARs while maintaining or increasing doses to the target (Figure 5). This strategy may reduce the risk of severe side effects. A previous study on X‐ray SBRT for pancreatic cancer^14^ has reached similar conclusions concerning the efficacy of plan adaptation. However, proton beams demonstrate a steep dose fall off, particularly beyond the distal edge of the tumor; thus, a treatment plan that is robust against daily anatomical variations needs to be created. Therefore, beam angles of 150° and 210° were selected in this study to minimize changes in water equivalent thickness along the beam path.^18^, ^19^

Doses to the OARs slightly increased even with DA in some cases. These increases were considered to be caused by optimization trade‐offs necessary to satisfy the constraints for the target and other OARs. However, except for one case (Patient 3), they remained within the tolerance limits. The constraint for the stomach_PRV could not be satisfied with DA in patient 3 because of its overlap with the GTV (Figure S1), making it challenging to meet the constraint while maintaining adequate target coverage. However, as the dose to the stomach itself satisfied the constraint with DA, the radiation oncologist deemed the plan clinically acceptable. This indicates that the stomach_PRV constraint may be of relatively lower clinical importance compared with the stomach itself, at least in this specific case.

However, the concept of a PRV margin cannot be entirely dismissed. DA can account for inter‐fractional anatomical variations, whereas intra‐fractional motion—particularly of the GI tract—is known to occur even over short time intervals.^9^ PRV margins of 3–5 mm for the GI tract have been applied during online adaptive radiation therapy.^31^, ^32^ The CT datasets in this study were not acquired in a treatment room; thus, applying a 5‐mm PRV margin is considered reasonable. Therefore, the use of a small PRV margin remains justified in general to ensure dosimetric robustness against intra‐fractional motion, although the stomach_PRV was not critical for clinical decision‐making in this particular case.

In this study, the pCT and dCT images used for treatment planning and DDE were acquired under end‐exhalation breath‐hold conditions and were not based on 4DCT. Irradiation in RGPT is performed with a gating window of ± 2 mm, leading to a residual motion of approximately ± 2 mm during beam delivery, which corresponds to the residual motion observed between two adjacent phases of a 4DCT. A previous study has validated the use of breath‐hold CT images for dose evaluation in RGPT^33^ and considered the use of breath‐hold CT for dose evaluation as appropriate in this study.

This study revealed the efficacy of plan adaptation in SBPT for pancreatic cancer, but it had several limitations. First, the patient cohort comprised only 10 individuals, which may be considered limited for broad generalizations, particularly considering the complex and variable anatomy of the abdominal region (Figure 4). Even with this limited sample size, our findings indicate that a daily adaptive strategy for SBPT in pancreatic cancer may be effective, particularly in mitigating GI toxicities and improving tumor control outcomes.

Second, the accuracy of DIR may affect the results of accumulated dose calculations. Previous studies that investigated DIR accuracy have reported that contour‐based DIR and hybrid DIR generally provide higher accuracy than the intensity‐based DIR utilized in this study, particularly in the abdominal region where low soft tissue contrast and GI gas are present.^34^, ^35^ However, the intensity‐based DIR has been acceptable for dose accumulation when the Dice similarity coefficient exceeds approximately 0.89.^35^ Further, other studies have used DIR‐based dose accumulation in clinical evaluation, indicating that despite inherent uncertainties, it remains clinically useful.^36^, ^37^

In this study, the decision regarding adaptive replanning relied on the dose distribution of each treatment day rather than on accumulated dose. Therefore, potential uncertainties associated with dose warping due to DIR are unlikely to directly affect the DA decisions. From this perspective, the adaptive strategy based on the proposed DDE is considered clinically reasonable.

Workflow and clinical workload are important considerations for the clinical implementation of adaptive radiation therapy. Contour modification, plan re‐optimization, and quality assurance must be performed in online adaptive radiotherapy during the treatment session, which may prolong the patient's treatment time. Conversely, offline adaptive radiotherapy is performed using a conventional workflow in which replanning is conducted separately from the treatment session. Glide–Hurst et al. reported that replanning in offline adaptive workflow may require several hours of additional planning work (approximately 7 h), although this process does not prolong the patient's treatment time on the treatment day.^38^

This study assumed an offline daily adaptive strategy, although online adaptive therapy would be preferable to consider anatomical changes. Online adaptive proton therapy remains not available on commercial machines; however, its implementation is technically feasible, as similar systems have already been realized in X‐ray therapy, including Ethos (Varian Medical Systems, Palo Alto, CA), and MR‐guided radiation therapy platforms, including Unity (Elekta, Stockholm, Sweden).^39^, ^40^ Therefore, online adaptive therapy is likely to be implemented in proton therapy in the future. The method proposed in this study enables proton adaptive therapy based on DDE and may facilitate the safe implementation of higher prescription doses.

## 5 CONCLUSIONS

This study aimed to quantitatively evaluate the efficacy of plan adaptation in SBPT for pancreatic cancer and to investigate the appropriate frequency of plan adaptation. Plan adaptation was required in more than 40% of fractions (21 out of 50) based on DDE, with an average of 2.1 adaptations per patient, in the 10 cases of SBPT for pancreatic cancer included in this study. This study indicated that performing DDE on daily CT images and applying plan adaptation could help maintain or improve target dose coverage while reducing doses to the OARs.

## AUTHOR CONTRIBUTIONS

Yuto Matsuo collected the data, made the simulation plans, and manuscript description. Yusuke Uchinami and Norio Katoh contoured targets and organs for all evaluated patients and verified them. Keiji Nakazato provised advises on the dose evaluation system. Seishin Takao and Naoki Miyamoto supervised this study. Yuto Matsuo, Seishin Takao, and Naoki Miyamoto interpreted the data. Takayuki Hashimoto, Taeko Matsuura and Hidefumi Aoyama reviewed the manuscript. All authors checked and approved the final draft for submission.

## CONFLICT OF INTEREST STATEMENT

The authors declare no conflicts of interest.

## Supporting information




**Supporting Information**: 2025‐08987‐sup‐0002‐SI_Table‐S01.docx

**Supporting Information**: 2025‐08987‐sup‐0003‐SI_Figure‐S01.pdf
