# Supplementary material for: Evaluation of the efficacy of plan adaptation in stereotactic body proton therapy for pancreatic cancer
Source: J Appl Clin Med Phys. 2026 May 28;27(5):e70644. doi: 10.1002/acm2.70644 (PMC13240328; doi:10.1002/acm2.70644)
Supplement: Supplementary file 1 — Supporting Information: 2025‐08987‐sup‐0002‐SI_Table‐S01.docx [file ACM2-27-e70644-s002.docx]

**Supplementary materials**

**Supplementary Table S1 Comparison of the DVH metrics between NA and DA**

| ROI | DVH metrics | median (range) | | |
| --- | --- | --- | --- | --- |
|  |  | NA | DA | *p*-value |
| CTV_EVAL | D_90_ | 38.0 (29.7-41.8) | 40.4 (29.7-45.3) | <0.05 |
|  | D_2_ | 49.0 (44.6-51.9) | 48.6 (45.0-51.9) | 0.1105 |
| CTV40 | D_99_ | 32.9 (17.1-38.1) | 35.0 (17.1-42.6) | <0.05 |
| GTV | D_50_ | 46.8 (37.5-48.5) | 46.8 (38.9-48.1) | 0.5941 |
| Stomach | V_33_ | 0.0 (0.0-1.0) | 0.0 (0.0-0.1) | <0.05 |
| Stomach_PRV | V_38_ | 0.0 (0.0-2.7) | 0.4 (0.0-1.1) | <0.05 |
|  | D_0.5_ | 36.2 (0.3-46.1) | 35.7 (0.3-47.0) | <0.05 |
| Duodenum | V_33_ | 0.0 (0.0-0.3) | 0.0 (0.0-0.0) | <0.05 |
| Duodenum_PRV | V_38_ | 0.0 (0.0-1.0) | 0.0 (0.0-0.4) | <0.05 |
|  | D_0.5_ | 28.7 (0.1-41.5) | 2.9 (0.1-38.4) | <0.05 |
| Small bowel | V_33_ | 0.0 (0.0-0.0) | 0.0 (0.0-0.0) | 1.000 |
| Small bowel_PRV | V_38_ | 0.0 (0.0-0.5) | 0.0 (0.0-0.4) | <0.05 |
|  | D_0.5_ | 19.2 (0.2-37.6) | 22.8 (0.2-35.3) | 0.4478 |
| Large bowel | V_33_ | 0.0 (0.0-1.6) | 0.0 (0.0-0.0) | <0.05 |
| Large bowel_PRV | V_38_ | 0.0 (0.0-4.4) | 0.0 (0.0-0.4) | <0.05 |
|  | D_0.5_ | 5.3 (0.1-48.3) | 11.4 (0.1-38.8) | <0.05 |

Abbreviations: ROI, region of interest; CTV, clinical target volume; GTV, gross tumor volume; PRV, planning at risk volume. Dx refers to the dose received by X percent of the target volume at least; Vx refers to volumes irradiated X Gy(RBE) at least.
