# Supplementary material for: Evaluation of the efficacy of plan adaptation in stereotactic body proton therapy for pancreatic cancer
Source: J Appl Clin Med Phys. 2026 May 28;27(5):e70644. doi: 10.1002/acm2.70644 (PMC13240328; doi:10.1002/acm2.70644)
Supplement: Supplementary file 2 — Supporting Information: 2025‐08987‐sup‐0003‐SI_Figure‐S01.pdf [file ACM2-27-e70644-s001.pdf]

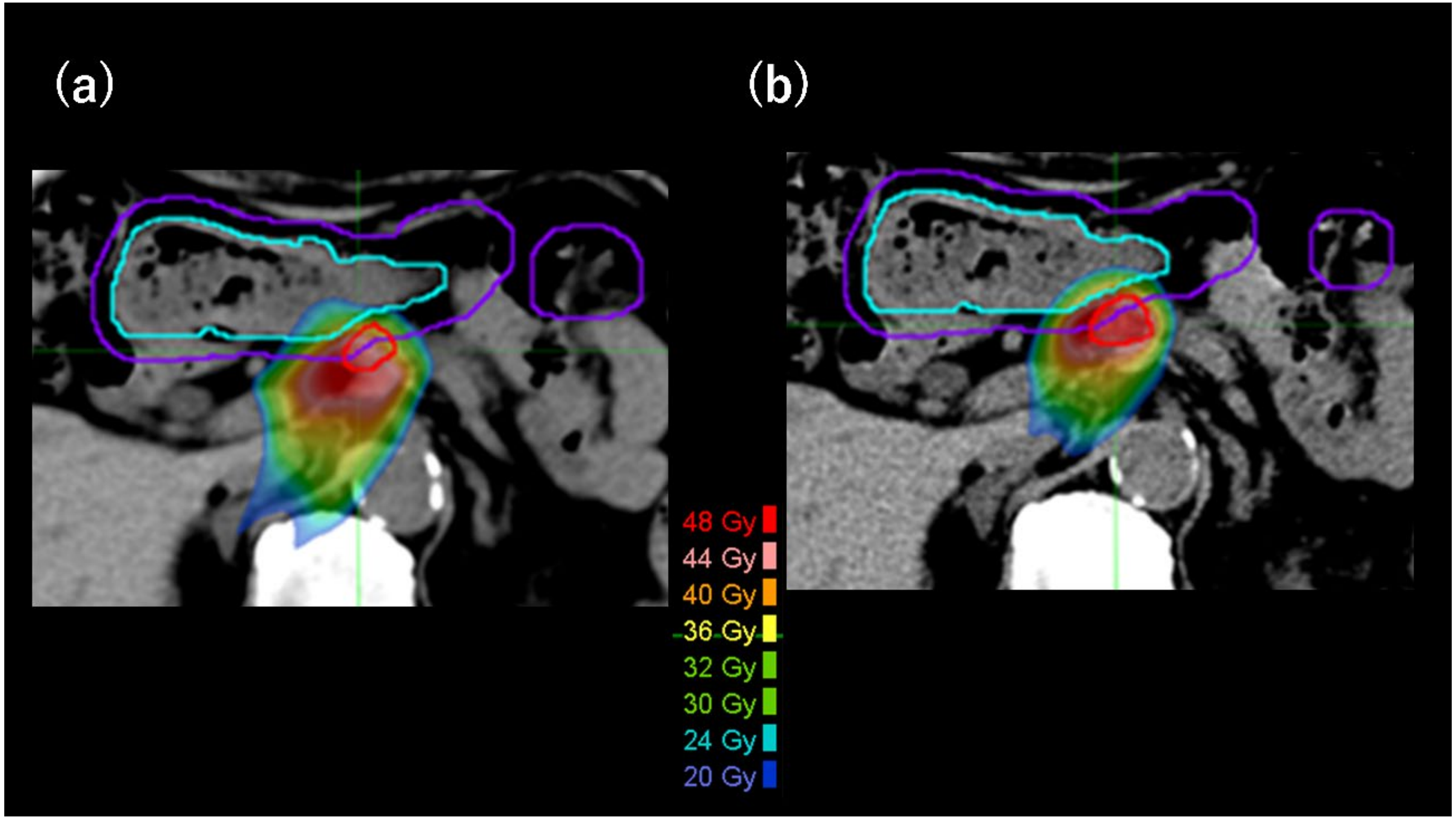

Supplementary Figure S1 Dose distribution of patient 3 on day 4

The red, cyan and purple line indicate GTV, stomach, and stomach_PRV, respectively.

(a) Forward calculation using latest plan on dCT

(b) Adaptive plan on dCT
